# Choice of reference measurements affects quantification of long diffusion time behaviour using stimulated echoes

**DOI:** 10.1002/mrm.26711

**Published:** 2017-05-03

**Authors:** Michiel Kleinnijenhuis, Jeroen Mollink, Wilfred W. Lam, Paul Kinchesh, Alexandre A. Khrapitchev, Sean C. Smart, Saad Jbabdi, Karla L. Miller

**Affiliations:** ^1^ Oxford Centre for Functional MRI of the Brain University of Oxford Oxford United Kingdom; ^2^ Department of Anatomy Radboudumc Nijmegen The Netherlands; ^3^ Sunnybrook Research Institute University of Toronto Toronto Canada; ^4^ Cancer Research UK & Medical Research Council Oxford Institute for Radiation Oncology Department of Oncology, University of Oxford Oxford United Kingdom

**Keywords:** stimulated echo, diffusion time, restriction, biexponential diffusion, apparent diffusion coefficient

## Abstract

**Purpose:**

To demonstrate how reference data affect the quantification of the apparent diffusion coefficient (ADC) in long diffusion time measurements with diffusion‐weighted stimulated echo acquisition mode (DW‐STEAM) measurements, and to present a modification to avoid contribution from crusher gradients in DW‐STEAM.

**Methods:**

For DW‐STEAM, reference measurements at long diffusion times have significant *b*_0_ value, because *b* = 0 cannot be achieved in practice as a result of the need for signal spoiling. Two strategies for acquiring reference data over a range of diffusion times were considered: constant diffusion weighting (fixed‐*b*_0_) and constant gradient area (fixed‐*q*_0_). Fixed‐*b*_0_ and fixed‐*q*_0_ were compared using signal calculations for systems with one and two diffusion coefficients, and experimentally using data from postmortem human corpus callosum samples.

**Results:**

Calculations of biexponential diffusion decay show that the ADC is underestimated for reference images with *b* > 0, which can induce an apparent time‐dependence for fixed‐*q*_0_. Restricted systems were also found to be affected. Experimentally, the exaggeration of the diffusion time–dependent effect under fixed‐*q*_0_ versus fixed‐*b*_0_ was in a range predicted theoretically, accounting for 62% (longitudinal) and 35% (radial) of the time dependence observed in white matter.

**Conclusions:**

Variation in the *b*‐value of reference measurements in DW‐STEAM can induce artificial diffusion time dependence in ADC, even in the absence of restriction. Magn Reson Med 79:952–959, 2018. © 2017 The Authors Magnetic Resonance in Medicine published by Wiley Periodicals, Inc. on behalf of International Society for Magnetic Resonance in Medicine. This is an open access article under the terms of the Creative Commons Attribution License, which permits use, distribution and reproduction in any medium, provided the original work is properly cited.

## INTRODUCTION

Dependence of the apparent diffusion coefficient (ADC) on diffusion time can reveal tissue properties with significance to neural health and disease. For instance, water molecules that are confined to a compartment exhibit reduced ADC with increased diffusion time. Long diffusion times can also probe membrane permeability. This dependence is often used in signal models that aim to improve biological specificity of diffusion MRI over nonspecific measures such as fractional anisotropy 1, 2, 3, 4, 5, 6, 7. To exploit these subtle changes in ADC, it is crucial that the measurements accurately reflect the diffusion propagator (ie, the displacement profiles over a range of diffusion times).

Diffusion time dependence has primarily been used to infer MRI measures of compartment size. Diffusion time dependence perpendicular to white matter tracts provides a marker of axon diameter, which has been proposed for improving tractography 8, 9, estimating conduction velocity 10, and monitoring of disease status 11. Long diffusion time signals can also characterize the mesoscopic organization of tissue 5, providing markers for axonal varicosities 12, undulations 13, and white matter fiber dispersion 14, 15. Finally, membrane permeability estimates based on diffusion time 6, 16 are a potential biomarker of apoptosis 17, tissue composition (eg, myelination 18), and tumor detection/classification 19, 20, 21.

Long diffusion time measurements face important technical challenges. Diffusion‐weighted spin‐echo (DW‐SE) measurements experience T_2_ signal loss that is intrinsically tied to long diffusion times and drastically reduces signal‐to‐noise ratio. Diffusion‐weighted stimulated echo acquisition mode (DW‐STEAM) 22 uncouples T_2_ signal loss from diffusion time and therefore may be more appropriate for long diffusion time measurements.

In this note, we demonstrate the importance of the sequence parameters for DW‐STEAM reference measurements, which are often referred to as *b* = 0, but in practice can have significant *b*‐value for long diffusion times. In particular, we show that these reference measurements can have a major effect on estimated ADCs for systems that exhibit non‐Gaussian diffusion, which is characteristic of all biological tissues. Crucially, including the *b*‐matrices in the ADC calculations is insufficient to draw valid conclusions about restrictions: The *b*‐values of both the diffusion‐weighted and reference measurements need to be kept constant over diffusion times.

## THEORY

### Diffusion Time Dependence of the ADC

In any diffusion‐weighted sequence, varying the diffusion time (Δ) between diffusion‐encoding gradients alters the diffusion‐weighting *b* if other parameters are kept constant:

(1) $$b=\gamma^{2}\delta^{2}G^{2}\left(\Delta -\frac{\delta }{3}\right)=\;q^{2}\left(\Delta -\frac{\delta }{3}\right).$$

Provided the displacement profile is Gaussian, diffusion measurements yield the same ADC, independent of the choice of *b* or Δ. However, with non‐Gaussian diffusion 23, 24, 25, 26, the choice of *b* is crucial for interpreting the diffusion time dependence of the ADC. For example, in biexponential diffusion—a simple departure from Gaussian diffusion—the ratio of a diffusion‐weighted signal, S(Δ), and a reference measurement, S_0_(Δ), is governed by

(2) $$\frac{S(\Delta )}{S_{0}(\Delta )}=fe^{-bD_{1}}+(1-f)e^{-bD_{2}}.$$

In this scenario, varying Δ with constant *q* (varying *b*) induces an apparent dependence of the ADC on diffusion time. This diffusion time dependence occurs for any protocol in which log(S/S_0_) is a nonlinear function of *b*, but would not be observed under constant *b* (varying *q* = γδG). Crucially, the need for constant *b* holds for both diffusion‐weighted *and* reference acquisitions with nonzero *b*‐value. This is particularly important for DW‐STEAM, in which reference measurements have significant *b*‐value as a result of the configuration of crusher gradients.

### DW‐STEAM.

**Figure 1:**
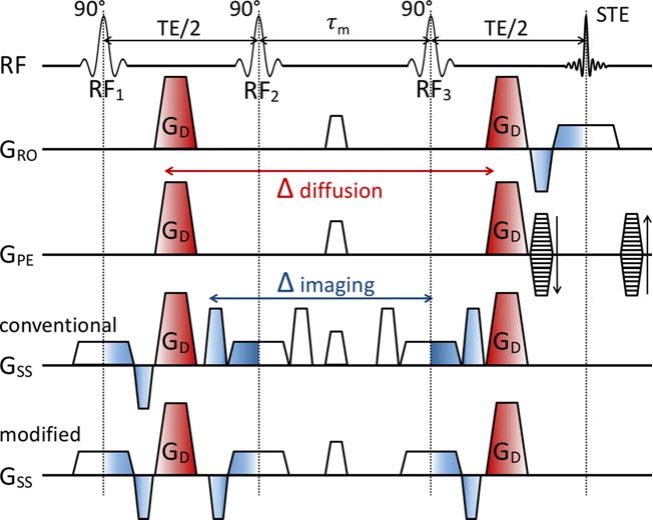
DW‐STEAM pulse sequence modification. The diffusion‐weighting effect of the diffusion‐encoding (red) and imaging gradients (blue) are indicated by color gradients. In the conventional scheme (fourth row), a pair of crushers is used on the slice‐select axis to remove unwanted signal from the third RF pulse. These crushers (and half of the slice‐select gradients) are separated by the mixing time τ_m_, resulting in a substantial diffusion weighting at long diffusion times (see Supporting Fig. S1). This diffusion weighting—if not properly handled—results in considerable variation in *b*‐value with diffusion time, as well as directional bias in the diffusion gradient directions 27. Because in the DW‐STEAM sequence, the diffusion‐encoding gradient already provides sufficient dephasing of the signal from RF3, the crushers are superfluous and can be replaced by usual slice‐select refocussing. The modified scheme (bottom row) effectively removes the contribution of the imaging gradients that builds up during the mixing time by flipping the two crusher gradients to form pairs with the slice‐select gradients to refocus the diffusion encoding outside of the mixing time. Adapted from Kinchesh et al. 28.

In DW‐SE, the range of diffusion times that can be probed is limited by T_2_ signal loss at long Δ. DW‐STEAM 22 is an attractive alternative that stores magnetization along the longitudinal axis during the mixing time (τ_m_) between the second and third 90 ° pulse (Fig. 1). DW‐STEAM incurs a twofold signal reduction, but exhibits the much slower T_1_ relaxation during τ_m_.

However, in DW‐STEAM the diffusion weighting from non‐diffusion‐encoding gradients can become substantial at long τ_m_ as a result of crushers that remove signal created by the third radiofrequency (RF) pulse (Fig. 1). In diffusion‐weighted images, various strategies have been proposed to account for these gradients, including the use of the full *b*‐matrix in analysis 29 and adapting the diffusion gradients 27. We propose a simple modification to the DW‐STEAM sequence to reduce diffusion weighting from the crusher gradient (Fig. 1, bottom row). By negating the polarity of the crusher and slice‐select gradients, the net gradient area is minimized during the mixing time. In the remainder of this work, we consider the effect of the residual gradient area in reference images on ADC quantification.

### Reference Images with Varying Diffusion Times

Calculating the ADC necessitates a reference measurement to divide out the signal contributions unrelated to the diffusion gradients. For DW‐STEAM, these contributions come from T_1_ and T_2_ decay. For measurements at multiple diffusion times, the variable T_1_ decay during the mixing times requires a separate reference image for each Δ.

**Figure 2:**
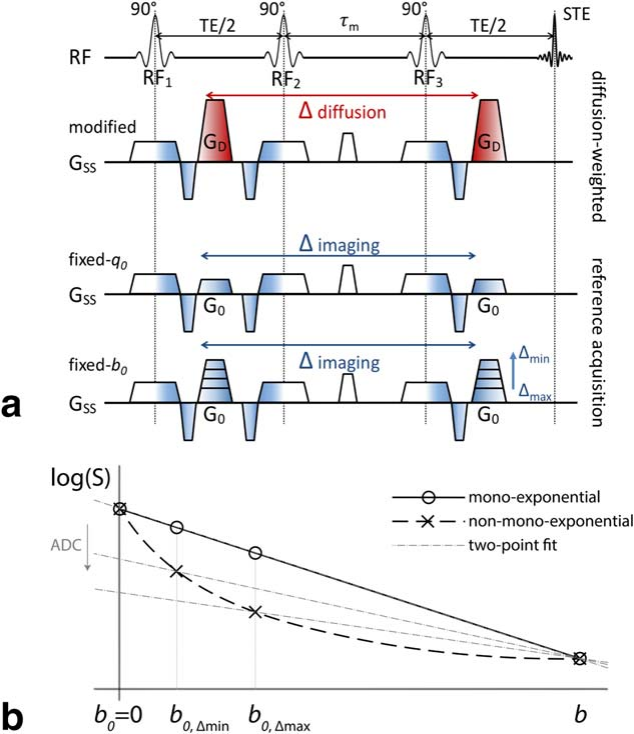
Reference measurements in the DW‐STEAM sequence. (**a**) DW‐STEAM reference measurements in which the gradient **G**_0_ (fixed‐*q*_0_; third row) or the *b*_0_‐value are kept constant (fixed‐*b*_0_; fourth row). In the DW‐STEAM sequence, the diffusion gradient (**G**_d;_ second row) is used as an imaging gradient in the reference measurements (denoted **G**_0_; applied over all three axes) to crush the signal from the third RF pulse. In the *b*_0_ image, the **G**_0_ is chosen according to the minimal *q*_0_‐value that achieves this, which might result in a nonnegligible *b*_0_‐value at long diffusion times. To obtain a constant *b*_0_‐value over diffusion times, the **G**_0_ needs to be increased for Δ < Δ_max_ compared with the minimal value G_0,Δmax_. (**b**) ADC calculation of signals undergoing mono‐exponential and non‐mono‐exponential decay. For mono‐exponential decay (solid line), the behavior is linear in *b*, and any two‐point fit of (*b, b*_0_) yields the same ADC. For the non‐mono‐exponential, the slope of the linear fit, and thus ADC, decreases if the *b*_0_‐value increases over the measurements.

Unlike DW‐SE, DW‐STEAM reference measurements accumulate significant *b*‐value at long diffusion times as a result of crusher gradients (Fig. 2a), which are smaller in amplitude than diffusion‐encoding pulses, but otherwise have similar properties, including separation by the mixing time.

If the crusher gradient moment is kept constant over diffusion times (fixed‐*q*_0_), the associated *b*‐value in the reference images (*b*_0_) increases with diffusion time. An alternate design is to keep the *b*_0_‐value constant over diffusion times (fixed‐*b*_0_), which requires varying the crusher area, *q*_0_. In this case, the minimum constant *b*_0_‐value over diffusion times is determined by *b*_0_ at the longest diffusion time *b*_0,Δmax_.

It is instructive to compare the fixed‐*b*_0_ and fixed‐*q*_0_ approaches for mono‐ and biexponential decay, in which the latter is a representative deviation from Gaussian diffusion that is not driven by restriction. The ADC from a DW‐STEAM experiment can be calculated from a two‐point fit as (Fig. 2b)

(3) $$ADC=\log\left(S/S_{0}\right)/\left(b-b_{0}\right).$$

For mono‐exponential decay, this calculation yields the same ADC independent of *b*_0_. When the decay is biexponential (Fig. 2b, dashed line), however, a two‐point fit yields an ADC that decreases with increasing *b*_0_ (Fig. 2b, dash‐dotted lines). Therefore, fixed‐*q*_0_ designs yield an artificial time‐dependence of the ADC as a result of the variation in *b*_*0*_‐value over diffusion times, which might be interpreted incorrectly as indicating restriction. Next we quantify this effect using simple simulations of unrestricted and restricted diffusion.

## METHODS

### ADC Calculations

The ADCs were compared between simulated measurements in which either *b*_0_ or *q*_0_ was held constant over diffusion times. For fixed‐*b*_0_, the *b*_0,Δmax_ was chosen as [10,20,30]% of the *b*‐value of the diffusion‐weighted images to reflect the range found in literature 12, 19, 27, 30, 31. The *b*_0_‐values are then

(4) $$\;\begin{array}{l} \begin{array}{cc} b_{0}\left(\Delta \right)=b_{0,\Delta_{max}} & \;\;\;\;\;\;\;\;\;\;\;\;\text{for}\;\text{fixed}‐b_{0}\;\;\text{and} \end{array}\\ \begin{array}{cc} b_{0}\left(\Delta \right)=q_{0,\Delta_{max}}{}^{2}\left(\Delta -\delta /3\right) & \text{for}\;\text{fixed}‐q_{0}\;\;\text{where} \end{array}\\ q_{0,\Delta_{max}}=sqrt(b_{0,\Delta_{max}}/\left(\Delta_{max}-\delta /3\right)). \end{array}$$

First, we evaluated the signal with and without the sequence modification (Supporting Fig. S1). Second, we investigated the influence of reference measurements on ADC estimates of a biexponential signal model and a biophysical tissue model. All calculations assume infinitely narrow pulses. Signal calculations were performed using the MISST toolbox 32, 33, 34 that uses the matrix formalism 35 to compute the signal for arbitrary pulse sequences.

The biexponential signal model is characterized by diffusion coefficients D_s_ (slow) and D_f_ (fast) and volume fraction *f*_s_. Biexponential decay was simulated for four fast diffusion coefficients relative to D_s_, given by D_f_ = [1,3,5,7] *×* D_s_. The first case (D_f_ = D_s_) represents mono‐exponential diffusion. The fast volume fraction *f*_s_ was set to 0.33 in accordance with literature 36. Signal simulations were conducted to probe regimes of normalized diffusion time Δ/Δ_max_ and contrast *b* ·D_s_ (*b*‐value in terms of signal contrast for the slow diffusion coefficient).

The biophysical model is a simple two‐compartment model with restricted intracellular diffusion in cylinders with radius *r*, diffusion coefficient D_i_ and volume fraction *f*_i_, and hindered diffusion in the extracellular space with diffusion coefficient D_h_. The evaluated ratios of the restricted and hindered diffusion coefficient were D_h_ = [1,0.75,0.50,0.25] *×* D_i_. D_i_ was set to 2 μm^2^/ms and *f*_i_ = 0.8, in keeping with the fraction found in white matter tissue 37. Experimental regimes investigated for this model were *b* = [0.0–8.5] ms/μm^2^ perpendicular to the cylinders and Δ = [10–500] ms.

### MRI Data Acquisition

Postmortem human corpus callosum samples from two brains provided by the Thomas Willis Brain Collection in Oxford were scanned. Coronal slabs of 5‐mm thickness were cut at the level of the anterior commissure. From each slab, a 45 *×* 25 mm block was cut, including the medial corpus callosum and parts of the centrum semiovale, cingulate, and superior frontal gyri. The blocks were soaked in phosphate‐buffered saline for 72 h and then transferred to a syringe filled with Fluorinert (3M, St. Paul, MN) 24 h before imaging.

MRI was performed on a 9.4 Tesla (T) 160‐mm horizontal bore VNMRS preclinical scanner (Varian Inc, Corona, CA) with maximum gradient of 400 mT/m and a 26‐mm ID quadrature birdcage coil (Rapid Biomedical GmbH, Germany). DW‐STEAM using a single‐line readout was adapted to minimize the effect of imaging gradients on diffusion weighting 28. Measurements at eight diffusion times Δ = [70,100,150,200,250,300,350,400] ms were acquired using 30 directions distributed over the sphere. The repetition time (TR) was minimized for each diffusion time: TR = [2.4,2.4,2.4,2.4,2.4,2.6,3.6,4.1] s, with a minimum of 2.4 s to allow T_1_ recovery between excitations (T_1_ over our sample was estimated to be approximately 600 ms). The effective echo time was 16 ms. Five slices were acquired at 0.4 *×* 0.4 *×*0.4 mm (matrix = 128 *×* 96; field of view = 51.2 *×* 38.4 mm). The complex data were resampled (matrix 64 *×* 48) and tapered with a Tukey window (α = 0.4) to boost SNR and reduce Gibbs ringing.

The diffusion‐weighted measurements used *b* = 3.5 ms·μm^−2^ with gradient duration δ = 2.22 ms and variable gradient strength |G| = 375–157 mT/m for Δ = 70–400 ms. For each Δ, two reference measurements were performed: one with constant *q*_0_ = 0.023 μm^−1^ as the minimal value that adequately spoilt the signal generated from the third RF pulse (corresponding *b*_0_ = 0.117–0.631 ms·μm^−2^) and one with constant *b*_0_ = 0.631 ms·μm^−2^ as the *b*_0_ value with *q*_0_ at Δ_max_ = 400 ms.

### MR Data Analysis

Diffusion tensors were fitted to the DW‐STEAM data using FDT from FSL 38. The full *b*‐matrix (ie, accounting for imaging gradients) was used to calculate the *b*‐values and *b*‐vectors. Tensor fitting was performed for each diffusion time separately, and for the two reference data sets separately (fixed‐*b*_0_ and fixed‐*q*_0_), while using the same diffusion‐weighted measurements. Masks were created by thresholding two diffusion tensor metrics as follows: fractional anisotropy at > 0.20 for a white matter mask (which avoided including crossing‐fiber voxels, such as in the centrum semiovale that had a lower fractional anisotropy); and mean diffusivity > 0.4 μm^2^/ms for a gray matter mask. The diffusivities are presented for the longitudinal (the first eigenvalue) and radial (the average of the second and third eigenvalues) directions.

## RESULTS

### ADC Calculations

**Figure 3:**
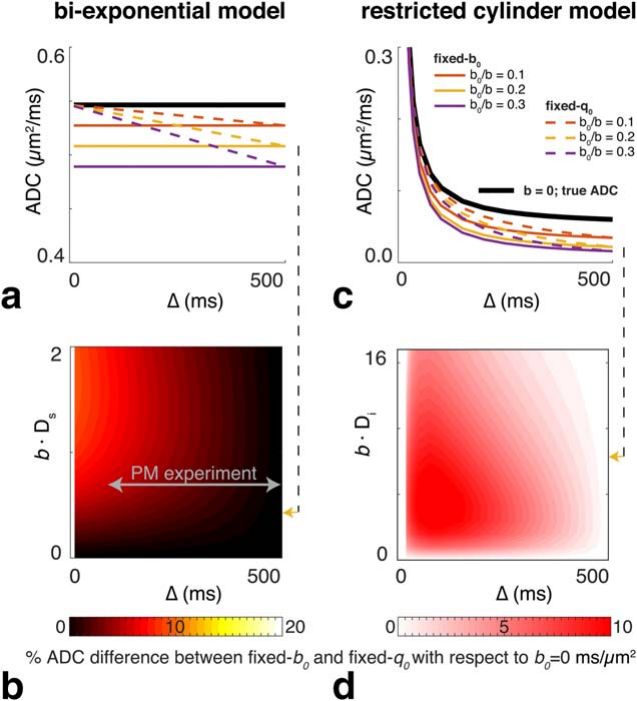
The effect of reference measurements on the ADC. The biexponential model is shown on the left (**a, b**), and the restricted cylinder model is shown on the right (**c, d**). (**a, c**) The line plots show specific examples for measurements with fixed‐*q*_0_ (dashed lines) versus fixed‐*b*_0_ (solid lines). Lines of different color show the behavior for different *b*_0_,Δmax values, where *b*_0_,Δmax = [0, 10, 20, 30]% of the *b*‐value. (**b, d**) The contour plots show differences in ADCs for the two reference measurement schemes; the color map represents the ADC difference between the fixed‐*q*_0_ ADC and the fixed‐*b*_0_ ADC as a percentage of the “true” ADC at *b*_0_ = 0 ms/μm^2^. (**a**) ADC behavior in biexponential signal model (without restriction) at *b × * D_s_ = 0.5 (*b* = 2.5 ms·μm^−2^; D_s_ = 0.2 μm^2^·ms^−1^, with D_f_ = 5 *×* D_s_ and *f*_s_ = 0.3. b)% ADC differences between fixed‐*b*_0_ and fixed‐*q*_0_ for the biexponential model for D_f_ = 5 *×* D_s_ and *b*_0_/*b* = 0.2. (**c**) ADC behavior in the restricted cylinder model at *b* = 4.0 ms·μm^−2^; D_i_ = D_h_ = 2.0 μm^2^·ms^−1^, with *r* = 5 μm and *f*_i_ = 0.80. d)% ADC differences between fixed‐*b*_0_ and fixed‐*q*_0_ for the restricted cylinder model for D_i_ = D_h_ and *b*_0_/*b* = 0.2. Note that the actual deviation from truth (*b*_0_ = 0 ms/μm^2^ measurement) is always maximal for fixed‐*b*_0_, because the *b*_0_‐value is determined by the longest diffusion time; however, our goal here is to quantify the difference between the two realistic measurement strategies: fixed‐*b*_0_ and fixed‐*q*_0_. The gray arrow indicates the regime of our postmortem measurements. All scales are linear. Supporting Figure S2 shows the effects of a different *b*_0_/*b* ratios and different diffusion coefficient ratios. The effect of varying the intracellular volume fraction and the cylinder radius is provided in Supporting Figures S3 and S4, respectively.

Figure 3a depicts mono‐exponential modeling of unrestricted, biexponential diffusion for the fixed‐*b*_0_ and fixed‐*q*_0_ schemes, with constant high *b*‐value across diffusion times and reference measurements with nonzero *b*_0_‐value. For fixed‐*b*_0_ (solid lines), the ADC is underestimated in comparison to a “true” *b*_0_ = 0 ms/μm^2^ (bold black line). Higher values for *b*_0,Δmax_ result in larger underestimation of the ADC. Nevertheless, fixed‐*b*_0_ does yield a *constant* ADC over diffusion times. The underestimation of the ADC also occurs for fixed‐*q*_0_ (dashed lines), but even more importantly, fixed‐*q*_0_ also induces an apparent diffusion time dependence in the absence of restriction, because the *b*_0_‐value increases with diffusion time.

The contour plot (Fig. 3b) quantifies, for an example regime *b*_0_/*b* = 0.2 and D_f_ = 5 *×* D_s_, the difference between the ADCs calculated with fixed‐*q*_0_ and fixed‐*b*_0_ measurements expressed as a percentage of the “true” *b*_0_ = 0 ms/μm^2^ (ie, ( ${\text{ADC}}_{\text{fixed}‐q_{0}}–{\text{ADC}}_{\text{fixed}‐b_{0}}$)/ADC*_b = 0_ ×* 100%). At low *b*‐values, the induced diffusion time dependence is small. However, a commonly used regime in experiments at long diffusion time, ie, *b × * D_s_≈[0.2–1.0] induces a large diffusion time dependence. The minimum *b*_0_‐value at the longest diffusion time and increasing difference in the biexponential signal components exacerbate the induced time dependence of the ADC (Supporting Fig. 2a). Conventional interpretations would incorrectly attribute the variation with diffusion time to restriction, despite this being a fundamentally unrestricted system.

In the case of genuine restriction (Figs. 3c and 3d), ADC decreases are observed that are similar in magnitude to the biexponential model for the ratio's D_h_/D_i_ that are common in these restriction models (D_h_ = [0.5–1] *×* D_i_). The differences between fixed‐*q*_0_ and fixed‐*b*_0_ decrease as the displacement profiles in the two compartments become more similar (eg, as D_h_ approaches the intracellular perpendicular ADC, as well as at short Δ where it even becomes negative as the diffusion in the intracellular space becomes free (D_i_ > D_h_)) (Supporting Fig. 2b). For any nonzero *b*_0_‐value (both fixed‐*b*_0_ and fixed‐*q*_0_), the signal attenuation curves differ from the “true” *b*_0_ = 0 ms/μm^2^, ultimately affecting the estimation of the model parameters.

### Postmortem MRI Data

**Figure 4:**
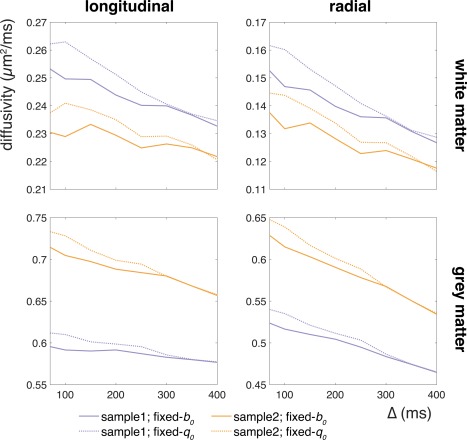
Differences in the diffusion time dependence of the ADC with two normalization strategies (fixed‐*q*_0_ and fixed‐*b*_0_). Panels show the parallel (left column) and perpendicular (right column) diffusivities averaged over voxels in a white matter (top row) and gray matter (bottom row) mask. The two samples are plotted in different colors. The diffusivity decreases with diffusion time for all cases, but in each case is exaggerated when fixed‐*q*_0_ normalization is used. Note that the diffusivities for both reference image sets converge at the longest diffusion time where the *b*_0_‐values for both reference measurement strategies are equal (however, these correspond to separate acquisitions, and thus the measurements are not identical).

Figure 4 depicts the diffusivities measured in two postmortem samples. Although the diffusivities differ between the samples, most likely because of differences in tissue preparation 39, 40, they follow similar trends. Diffusivity decreases are observed with increasing diffusion time for both longitudinal and radial directions, as well as in different tissue types (white and gray matter). This diffusion time dependence is exaggerated when the signal is normalized by the fixed‐*q*_0_ measurement compared with the fixed‐*b*_0_ measurement: The slope for fixed‐*q*_0_ is larger than for fixed‐*b*_0_. Differences in diffusion time dependence between fixed‐*b*_0_ and fixed‐*q*_0_ are quantified in Table 1.

**Table 1:** ADC Differences between Fixed‐*q*_0_ and Fixed‐*b*_0_ Strategies

ΔADC (%)	Fixed‐*q*_0_	Fixed‐*b*_0_	Fixed‐*q*_0_ effect
White matter longitudinal	7–11	4–8	44–80
White matter radial	19–20	15–17	35–35
Gray matter longitudinal	6–10	3–8	32–86
Gray matter radial	14–17	11–15	20–28

Note: The values in columns 1 and 2 indicate the difference in ADC between the short diffusion time (Δ = 70 ms) and the long diffusion time measurement (Δ = 400 ms), expressed as a percentage of the short diffusion time measurement. The third column shows the percentage of the ADC dependence as observed under fixed‐*b*_0_, which is added by dependence induced by fixed‐*q*_0_ measurements. The ranges indicate values for the two samples.

## DISCUSSION

We demonstrate that the choice of acquisition parameters for the reference *b*_0_‐image is essential for interpreting ADC measurements using DW‐STEAM. In a system that can be characterized by multiple diffusion coefficients, reference measurements with nonzero *b*‐value lead to underestimation of the “composite” ADC. As this underestimation increases with *b*_0_‐value, the common approach of using a constant crusher gradient strength for the reference measurement at each diffusion time induces an artificial diffusion time dependence. Therefore, in experiments that rely on subtle variations of the ADC over a long interval of diffusion times, incorrect normalization could lead to misinterpretation of the results.

The size of this induced diffusion time dependence depends on the tissue's diffusion properties and the sequence parameters. In simulations, we demonstrated that stronger deviation from mono‐exponential decay and a larger *b*_0_‐value for the reference measurement induces a larger diffusion time dependence. For typical acquisition parameters, the artificial effect can be on the order of the diffusion time dependence as a result of restrictions (Table 1, column 3). Data acquired from postmortem corpus callosum samples indicated that a considerable portion of the diffusion time dependence observed using fixed‐*q*_0_ references is consistent with multiple diffusion coefficients in the system. Nonetheless, the ADC still exhibited decreases with diffusion time under fixed‐*b*_0_ references, both perpendicular and parallel to the bundle, suggesting true restriction effects.

Although most studies investigating diffusion time dependence of ADC measures report a fixed *b*‐value, reporting of the *b*_0_‐values in DW‐STEAM is often omitted or inaccurate (ie, reported as *b* = 0 ms/μm^2^ or “non‐diffusion weighted”). The present investigation demonstrates that using the *b*‐matrix is only sufficient to account for this effect when the underlying diffusion process is well characterized by a single diffusion coefficient. Both *b* and *b*_0_ must be kept constant to draw conclusions about restriction. As a case in point, the use of fixed‐*b*_0_ measurements has changed our original interpretations of the data in Figure 4, which aimed to investigate diffusion time dependence along white matter fibers. There is not very strong evidence for a diffusion time dependence of the ADC in the longitudinal direction when a fixed‐*b*_0_ is used, whereas the fixed‐*q*_0_ data appear to suggest a small but consistent dependence.

In our simulations, a biexponential signal was generated to evaluate the effects of DW‐STEAM reference measurements in the presence of non‐Gaussian diffusion, which is well established in brain tissue 16. However, the choice of a biexponential system in our simulations is motivated by its simplicity and not to imply that this is the most appropriate tissue model. Our results extend to any form of non‐mono‐exponential signal decay.

The introduction of diffusion time dependence through variation in the *b*‐values is limited to situations in which two‐point fits are used to calculate a summary ADC from measurements with varying diffusion time. A model fit (eg, kurtosis (44), biexponential (45), CHARMED (46), AxCaliber (47)) to the nonnormalized data could be used to extract accurate and informative quantities from DW‐STEAM measurements, although these models would have to be adapted to account for T_1_ decay during the mixing time.

For models that rely on accurate estimation of ADC values (12,48), it becomes important to carefully consider the measurement regime, as not all regimes are equally affected. The exact sampling locations of the reference and diffusion‐weighted measurements along the decay curve determine the extent of the induced diffusion time dependence. In the low‐contrast regime, the effect is small: The linear extrapolation to the *b* = 0 ms/μm^2^ intercept is reasonably accurate. Higher *b*_0_‐values for the reference measurement at the longest diffusion time spread the sampled points for fixed‐*q*_0_ design over a larger range of the decay curve, and therefore result in a larger induced diffusion time dependence (Fig. 2).

Although these effects were demonstrated in postmortem tissue, the results presented here should apply to in vivo data. Postmortem tissue has been shown to exhibit similar diffusion time dependence to in vivo tissue (49). Our measurements had a slight residual variation in the *b*‐value with Δ and gradient direction (*b*_min_ = 3.39 ms·μm^−2^ at Δ = 70 ms quadratically increasing to *b*_max_ = 3.45 ms·μm^−2^ at Δ = 400 ms along the slice‐select axis). Considering the relatively small size of the *b*‐value variation in comparison to the *b*_0_‐variation and the use of the same diffusion‐weighted data in both analyses, this does not invalidate our fixed‐*b*_0_ versus fixed‐*q*_0_ comparison and conclusions. Another remaining issue is the directional bias 27 caused by the particular direction of the diffusion weighting in the *b*_*0*_‐image, with respect to all directions in the shell. One solution for this would be to acquire a reference image for each direction and diffusion time at the cost of significant increase in scan time.

## CONCLUSIONS

Future experiments with the DW‐STEAM sequence using variable diffusion times should be designed to avoid variable *b*‐values in the reference measurements to draw valid conclusions about quantities related to restriction, such as length scales and permeability. Incorporating the full *b*‐matrix is insufficient: To avoid confounding the effects of restriction and possible multiple diffusion coefficients in the system under study, it is important to keep the *b*‐value as well as the *b*_0_‐value constant over diffusion times. Unlike reference measurements with fixed‐*q*_0_, use of a constant *b*_*0*_‐value does not induce an apparent diffusion time dependence in the absence of restrictions. Fixed‐*b*_*0*_ is therefore preferable in experiments that draw conclusions from the relation between the ADC and diffusion time.

## Supporting information


**Fig. S1**. DW‐STEAM sequence modification efficacy. Resultant *b*‐values for conventional and modified DW‐STEAM sequence according to the postmortem protocol are shown in the top row. All gradients were applied in the slice‐select direction with the *b*‐value from the diffusion gradient set to *b*_diff_ = 3.5, *b*_diff_ = 0.7 (fixed‐*b*_0_) and *b*_diff_=0.0 ms·μm^−2^ over all diffusion times, as well as one in which the gradient was kept constant over diffusion times (fixed‐*q*_0_). As measured in the (worst‐case) direction along the slice‐select axis, the conventional implementation of the DW‐STEAM sequence results in a large range of *b*‐values over the diffusion times in our postmortem protocol. The *b*‐value accumulated from the imaging gradients alone is given by the blue line. The bottom row shows the ADC for conventional and modified DW‐STEAM. For these calculations, we used values for the parameters of the biexponential signal model close to those found in our postmortem samples (ie, D_s_ = 0.2, D_f_ = 1.0; f_s_ = 0.33), as well as *q*‐values for crusher and slice‐select gradients representative for our postmortem MR protocol (ie, *q*_c_ = *q*_s_ = 0.046 μm^−1^). For the conventional sequence implementation, the undesired *b*‐value variation implies considerable variation of the calculated ADC in the absence of restriction. The sequence modification, however, prevents the variation in *b*‐value and results in a flat ADC over diffusion times if the *b*‐value and *b*_0_‐value are constant. Next to demonstrating that the sequence modification is adequate, this indicates the importance of keeping the *b*‐value and *b*_0_‐value of the diffusion‐weighted images constant with diffusion time.
**Fig. S2**. Effect of reference measurements on the ADC. (a) Biexponential signal model (without restriction) with *f*_s_ = 0.33. (b) Restricted cylinder model with D_i_ = 2 μm^2^/ms, *f*_i_ = 0.80, and *r* = 5 μm. In each panel, the columns show the results of different ratios of the diffusion coefficients in the respective models. The line plots in the top row of each panel show specific examples for measurements with fixed‐*q*_0_ (dashed lines) and fixed‐*b*_0_ (solid lines). Lines of different color show the behavior for different *b*_0,Δmax_ values, in which *b*_0,Δmax_ = [0, 10, 20, 30] % of the *b*‐value. The three rows of contour plots show differences in ADCs for the two reference measurement schemes, where each row shows the results for a different *b*_0_/*b* ratio. The color map represents the ADC difference between the fixed‐*q*_0_ ADC and the fixed‐*b*_0_ ADC as a percentage of the “true” ADC at *b*_0_ = 0 ms/μm^2^. The top row in (a) plots the ADC versus Δ curves for *b × * D_s_ = 0.5 (*b* = 2.5 ms·μm^−2^; D_s_ = 0.2 μm^2^·ms^−1^, with D_f_ = 5 *×* D_s_ and *f*_s_ = 0.33) (ie, representative of our postmortem experiment). A larger difference between diffusion coefficients leads to increased underestimation of the ADC (with respect to the “true” *b*_0_ = 0 ms/μm^2^), as does a larger *b*_0_/*b* ratio. This is further quantified in the contour plots, where the effect of contrast parameter *b × * D_s_ can be examined. In (b) the top row shows the ADC versus Δ curves for *b* = 4.0 ms·μm^−2^, with *r* = 5 μm and *f*_i_ = 0.80. Here, the difference between the fixed‐*b*_0_ and fixed‐*q*_0_ decreases with increasing difference between the compartment diffusion coefficients, because the intracellular compartment is highly restricted and the ADC_i_ is low. As D_h_ decreases, the compartmental ADCs are more similar and the differences between fixed‐*b*_0_ and fixed‐*q*_0_ diminish. For very short Δ, in which diffusion approaches free diffusion in the intracellular compartment, the % ADC difference measure becomes negative when D_i_ > D_h_. Note that the actual deviation from truth (*b*_0_ = 0 ms/μm^2^ measurement) is always maximal for fixed‐*b*_0_, because the *b*_0_‐value is determined by the longest diffusion time; however, our goal here is to quantify the difference between the two realistic measurement strategies: fixed‐*b*_0_ and fixed‐*q*_0_. Different columns show the effect of a larger difference between the diffusion coeffiecients. The gray arrow indicates the regime of our postmortem measurements. All scales are linear. The effect of varying the intracellular volume fraction and the cylinder radius is provided in Supporting Figures S3 and S4, respectively.
**Fig. S3**. The effect of varying volume fraction on the ADC in the presence of multiple diffusion coefficients. (a) The ADC versus Δ curves (top row) and % ADC differences between fixed‐*b*_*0*_ and fixed‐*q*_*0*_ for the biexponential model with D_f_ = 5 × D_s_. The biexponential model exhibits high sensitivity to volume fraction variation, because it severely alters the ADC differences between the two pools (fast and slow). (b) ADC versus Δ curves (top row) and % ADC differences between fixed‐*b*_*0*_ and fixed‐*q*_*0*_ for the restricted cylinder model with D_h_ = D_i_ and r = 5 μm. Although the volume fraction changes the ADC, the relative difference between fixed‐*b*_*0*_ and fixed‐*q*_*0*_ are found to be very similar.
**Fig. S4**. The effect of cylinder radius on the ADC in the presence of multiple diffusion coefficients in the restricted cylinder model.
