# Supplementary material for: Choice of reference measurements affects quantification of long diffusion time behaviour using stimulated echoes
Source: Magn Reson Med. 2017 May 3;79(2):952–9. doi: 10.1002/mrm.26711 (PMC5811793; doi:10.1002/mrm.26711)
Supplement: Supplementary file 1 — Fig. S1. DW‐STEAM sequence modification efficacy. Resultant b‐values for conventional and modified DW‐STEAM sequence according to the postmortem protocol are shown in the top row. All gradients were applied in the slice‐select direction with the b‐value from the diffusion gradient set to b diff = 3.5, b diff = 0.7 (fixed‐b 0) and b diff=0.0 ms·μm−2 over all diffusion times, as well as one in which the gradient was kept constant over diffusion times (fixed‐q 0). As measured in the (worst‐case) direction along the slice‐select axis, the conventional implementation of the DW‐STEAM sequence results in a large range of b‐values over the diffusion times in our postmortem protocol. The b‐value accumulated from the imaging gradients alone is given by the blue line. The bottom row shows the ADC for conventional and modified DW‐STEAM. For these calculations, we used values for the parameters of the biexponential signal model close to those found in our postmortem samples (ie, Ds = 0.2, Df = 1.0; fs = 0.33), as well as q‐values for crusher and slice‐select gradients representative for our postmortem MR protocol (ie, q c = q s = 0.046 μm−1). For the conventional sequence implementation, the undesired b‐value variation implies considerable variation of the calculated ADC in the absence of restriction. The sequence modification, however, prevents the variation in b‐value and results in a flat ADC over diffusion times if the b‐value and b 0‐value are constant. Next to demonstrating that the sequence modification is adequate, this indicates the importance of keeping the b‐value and b 0‐value of the diffusion‐weighted images constant with diffusion time. Fig. S2. Effect of reference measurements on the ADC. (a) Biexponential signal model (without restriction) with f s = 0.33. (b) Restricted cylinder model with Di = 2 μm2/ms, f i = 0.80, and r = 5 μm. In each panel, the columns show the results of different ratios of the diffusion coefficients in the respective models. The line plots [file MRM-79-952-s001.docx]

Supporting Figure S1. DW-STEAM sequence modification efficacy.

Resultant *b*-values for conventional and modified DW-STEAM sequence according to the post mortem protocol are shown in the top row. All gradients were applied in the slice-select direction with the *b*-value from the diffusion gradient set to *b*_diff_=3.5, *b*_diff_=0.7 (fixed-*b_0_*) and *b*_diff_=0.0 ms⋅μm^-2^ over all diffusion times, as well as one where the gradient was kept constant over diffusion times (fixed-*q_0_*). As measured in the (worst-case) direction along the slice-select axis, the conventional implementation of the DW-STEAM sequence results in a large range of *b*-values over the diffusion times in our post-mortem protocol. The *b*-value accumulated from the imaging gradients alone is given by the blue line.

The bottom row shows the ADC for conventional and modified DW-STEAM. For these calculations, we used values for the parameters of the bi-exponential signal model close to those found in our post-mortem samples, i.e. D_s_=0.2, D_f_=1.0; f_s_=0.33, as well as *q*-values for crusher and slice-select gradients representative for our post mortem MR protocol, i.e *q*_c_=*q*_s_=0.046 μm^-1^. For the conventional sequence implementation, the undesired *b*-value variation implies considerable variation of the calculated ADC in the absence of restriction. The sequence modification, however, prevents the variation in *b*-value and results in a flat ADC over diffusion times if the *b*-value and *b_0_*-value are constant. Next to demonstrating that the sequence modification is adequate, this indicates the importance of keeping the *b*-value and *b_0_*-value of the diffusion-weighted images constant with diffusion time.

Supporting Figure S2. The effect of reference measurements on the ADC.

a) bi-exponential signal model (without restriction) with *f*_s_ = 0.33. b) restricted cylinder model with D_i_ = 2 μm^2^/ms, *f*_i_ = 0.80 and r=5 μm. In each panel, the colums show results of different ratios of the diffusion coefficients in the respective models. The line plots in the top row of each panel show specific examples for measurements with fixed-*q_0_* (dashed lines) and fixed-*b_0_* (solid lines). Lines of different colour show the behaviour for different *b_0_*_,Δmax_ values, where *b_0_*_,Δmax_ = [0, 10, 20, 30] % of the *b* value. The three rows of contour plots show differences in ADCs for the two reference measurement schemes, where each row shows the results for a different *b_0_*/*b* ratio. The colourmap represents the ADC difference between the fixed-*q_0_* ADC and the fixed-*b_0_* ADC as a percentage of the ‘true’ ADC at *b_0_*=0 ms/μm^2^.

In a) the top row plots the ADC vs. Δ curves for *b*⋅D_s_=0.5 (*b* = 2.5 ms⋅μm^-2^; D_s_ = 0.2 μm^2^⋅ms^-1^, with D_f_=5⋅D_s_ and *f*_s_ = 0.33), i.e. representative of our post mortem experiment). A larger difference between diffusion coefficients leads to increased underestimation of the ADC (with respect to the ‘true’ *b_0_*=0 ms/μm^2^), as does a larger *b_0_*/*b* ratio. This is further quantified in the contour plots, where the effect of contrast parameter *b*⋅D_s_ can be examined. In b) the top row shows the ADC vs. Δ curves for *b*=4.0 ms⋅μm^-2^, with *r*=5 μm and *f*_i_ = 0.80. Here, the difference between the fixed-*b_0_* and fixed-*q_0_* decreases with increasing difference between the compartment diffusion coefficients, because the intracellular compartment is highly restricted and the ADC_i_ is low. As D_h_ decreases, the compartmental ADCs are more similar and the differences between fixed-*b_0_* and fixed-*q_0_* diminish. For very short Δ, where diffusion approaches free diffusion in the intracellular compartment, the %ADC difference measure becomes negative when D_i_>D_h_.

Note that the actual deviation from truth (*b_0_*=0 ms/μm^2^ measurement) is always maximal for fixed-*b_0_* because the *b_0_*-value is determined by the longest diffusion time; however, our goal here is to quantify the difference between the two realistic measurement strategies fixed-*b_0_* and fixed-*q_0_*. Different columns show the effect of a larger difference between the diffusion coeffiecients. The grey arrow indicates the regime of our post-mortem measurements. All scales are linear. The effect of varying the intracellular volume fraction and the cylinder radius is provided in Supporting Figures S3 and S4, respectively.

Supporting Figure S3. The effect of varying volume fraction on the ADC in the presence of multiple diffusion coefficients. a) ADC vs. Δ curves (top row) and % ADC differences between fixed-*b_0_* and fixed-*q_0_* for the bi-exponential model with D_f_=5⋅Ds. The bi-exponential model exhibits high sensitivity to volume fraction variation, because it severely alters the ADC differences between the two pools (fast and slow). b) ADC vs. Δ curves (top row) and % ADC differences between fixed-*b_0_* and fixed-*q_0_* for the restricted cylinder model with D_h_=D_i_ and *r*=5 μm. Although the volume fraction changes the ADC, the relative difference between fixed-*b_0_* and fixed-*q_0_* are found to be very similar.

Supporting Figure S4. The effect of cylinder radius on the ADC in the presence of multiple diffusion coefficients in the restricted cylinder model.
